# “Deoxy” to be or “Desoxy” not to be—a century-old tale in the history of DNA nomenclature

**DOI:** 10.1128/jb.00401-23

**Published:** 2024-01-31

**Authors:** Gaurav Sharma, Jaimin Chodvadiya, Indranil Malik

**Affiliations:** 1 Department of Biotechnology, Indian Institute of Technology Hyderabad, Sangareddy, Telangana, India; Geisel School of Medicine at Dartmouth, Hanover, New Hampshire, USA

**Keywords:** DNA, deoxyribonucelic acid, desoxyribonucleic acid, nomenclature, history

## Abstract

This commentary discusses a comprehensive history of the first-ever use of pertinent words directly related to DNA, such as desoxyribose, deoxyribose, desoxyribonucleic acid, and deoxyribonucleic acid. With almost 100 years of the identification and nomenclature of desoxyribose sugar and desoxyribonucleic acid, the term “desoxy” continues to see limited use. We hope that whenever young researchers come across the sporadic occurrence of “desoxy” in any published text, they will not consider it a mistake.

## COMMENTARY

With time, evolution, and the mushrooming of “keyword” usage in modern science, many scientific terminologies have gone obsolete or archaic. Sometimes, a few archaic words swing by in the literature, causing a little bewilderment to the young generation of scientists like us. In such cases, primarily, we either disregard them for a while or do a quick follow-up to know their appropriateness. Astoundingly, not even one of the most revered terms in biology, i.e., deoxyribonucleic acid (DNA), is exempt from this archaism.

**Fig 1:**
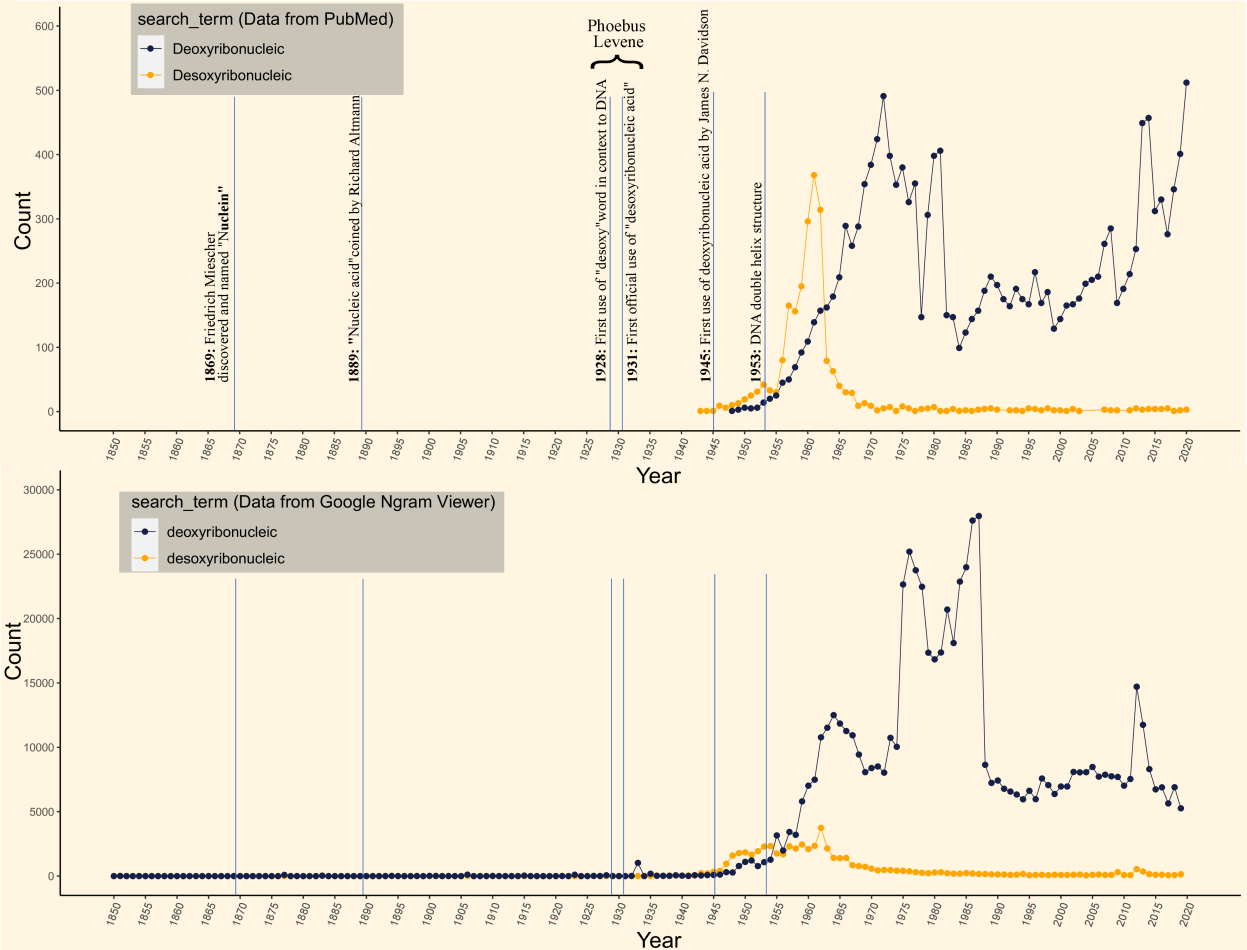
Dot charts showcasing the word-usage patterns of “desoxyribonucleic” and “deoxyribonucleic” terms in PubMed and Google Ngram Viewer: A few historic events have also been depicted on the timescale.

While reading a wonderfully compiled microbiology-history-oriented review by Prof. Roberto Kolter (1), we stumbled upon the term “desoxyribose” used in place of “deoxyribose,” which we felt was a “typo.” Following a quick search and further reading, our mistake turned into the realization that “deoxyribonucleic acid” was once referred to as “desoxyribonucleic acid,” which was eventually lost in the early days of modern molecular biology. To understand the relative usage of the words “desoxyribonucleic” and “deoxyribonucleic” over time, we analyzed two commonly used databases, i.e., Google Ngram Viewer (for books) and PubMed (for publications), and observed that “desoxyribonucleic” is still being used in several manuscripts and books (Fig. 1). Furthermore, the graph also shows that “desoxyribonucleic” was used exceedingly between 1930 and 1955, and later, “deoxyribonucleic” became the preferred term. Given the potential information bias for old records in the current index journals and the occasional absence of archive sections in their databases, we conducted a comprehensive investigation among the independent internet sources and targeted journals to unearth the initial usage of these two words.

The history of the present DNA goes back to 1869 when Friedrich Miescher coined the term Nuclein for a substance, which was the digested product of the lymphocytes isolated from the hospital bandages; this was published 2 years later (2). In 1889, Richard Altmann suggested that Nuclein should refer to a combination of nucleic acid and protein, and he used the term “nucleic acid” for the substance remaining after removal of all protein from nuclein (3, 4). Up to the early decades of the last century, two kinds of nucleic acids were known, one of which was initially termed “thymonucleic acid” (now known as DNA) with seemingly apposite prudence as it was obtained from the thymus gland and contains the pyrimidine base “thymine,” and the second was known as “ribonucleic acid” (RNA). The earliest use of the “Desoxy” word in the scientific literature in relation to “des-/deoxyribonucleic acid” can be traced back to the report on “structure of thymonucleic acids” by Phoebus Levene in his 1928 Paper with Efim London (5) and later in 1929 commentary in the Journal of Biological Chemistry (JBC) (6) mentioning a specific word “guaninedesoxypentoside,” which was the guanine nucleoside of desoxyribonucleic acid. They discovered that irrespective of being a reducing substance, this desoxysugar, a hydrolysis byproduct of these nucleosides, does not yield an osazone with phenylhydrazine. This is attributed to the absence of a 2′-hydroxyl group, in contrast to the well-documented ribose sugars, rendering it a fascinating component within the presently recognized DNA structure. Later in 1930, Levene and Dillon used the word desoxyribophosphoric acid in their JBC paper focused on intestinal nucleotidase (7); this can be considered the first synonymous usage of the des-/deoxyribonucleic acid. In the same year, Levene and Jorpes also used “desoxypentose nucleic acid” for thymonucleic acid in their JBC article (8). However, it was in 1931 that the exact term “desoxyribonucleic acid” was used in one of the notable books, Nucleic Acids: American Chemical Society, No. 56, by Levene, Bass, and Noyes (9). Following this, the term “desoxyribonucleic acid” was frequently used in the next two decades.

Interestingly, until the 1940s, it was believed that ribonucleic acid was present only in plants, bacteria, and fungi and desoxyribonucleic acid in animal cells. Challenging that view, Jean Brachet (1940) followed by James N. Davidson (1943) demonstrated that ribonucleic acid is also abundantly present in animal tissues such as the liver, pancreas, and brain as compared to “desoxyribonucleic acid” (10). Moreover, Davidson JN also suggested that DNA is present in the nucleus, and RNA is more abundant in the cytoplasm. In an arising series of terminology debates (11–13), “desoxyribonucleic acid” and “ribonucleic acid” were proposed to be renamed as “chromonucleic acid” and “plasmonucleic acid,” respectively, based on their relative presence in nuclear chromatin and plasmosome (cytoplasm). Amusingly, in 1949, it was also proposed to address “desoxyribonucleic acid” as “doRNA” (14). However, to date, it is still unclear how thymonucleic acid became desoxyribonucleic acid, and more importantly, how the field arrived at “desoxy” not to be and “deoxy” to be.

The term “desoxyribonucleic acid” was frequently used at least until 1945 when Davidson and Waymouth used the term “deoxyribonucleic acid” in a Biochemical Journal article (15, 16). Later, in 1949, the abbreviation “DNA” was used by Davidson, Leslie, and Waymouth in their Biochemical Journal article (17). Seemingly, there is no single report or viewpoint that suggested the official transition from “desoxy” to “deoxy.” Surprisingly, until 1944, Davidson regularly used “desoxyribonucleic acid” in all his manuscripts (18, 19); however, in 1945, it changed into deoxyribonucleic acid. In another similar development, Henry, Stacey, and Teece had also used the term “deoxyribonucleic acid” in their 1945 Nature letter (20) in contrast to their 1943 Nature letter using “desoxyribonucleic acid” (21) while discussing the histochemistry of the Gram-staining reaction in microorganisms. Other notable researchers, such as Chargaff, Watson, Crick, and Wilkins, also opted for the contrasting usage of these terms. Erwin Chargaff used the term “desoxyribonucleic acid” in all his publications up to 1953 and changed it to “deoxyribonucleic acid” after that. Watson and Crick in their landmark paper (22) used the term “deoxyribonucleic acid,” which, intuitively enough, started dominating ever since (Fig. 1). However, one must notice that out of the seven references in that paper, four used “desoxyribonucleic acid” including Wilkins and colleagues’ 1953 Biochim Biophys Acta paper (23). Contrary to this, Wilkins and colleagues also published an article in 1953 (24) along with Watson and Crick’s landmark 1953 paper where they used the term “deoxyribonucleic acid.” These examples suggest the interchangeable usage between desoxy and deoxy just before “deoxyribonucleic acid” became the predominant term.

Overall, it can be argued that although both “desoxyribonucleic acid” and “deoxyribonucleic acid” have no literal difference in meaning, there might be a hidden tale behind their usage. Most of the DNA history takes a sharp turn from Friedrich Miescher (isolation of Nuclein in 1869) (2) to Albrecht Kossel (discovery of five nucleotide bases) (25) to Avery, Macleod, and McCarty (DNA-mediated bacterial transformation suggesting it as a genetic material in 1944) (26). Although there was a switch from “desoxy” to “deoxy” in 1945, we were not able to identify any assertive reason behind this. Prof. Roberto Kolter and one of the reviewers of this article provided a prevailing argument suggesting that the “lingua franca” of chemistry in the 19th century was German; considering this, English speakers learned such words in German first, and therefore, their initial pronunciation and spellings retained the “s” in “desoxy”; however, later, slowly migrated to the version without the “s” as in “deoxy.” Even now, DNA is read/spoken/written as Desoxyribonukleinsäure (in German) and Ácido Desoxirribonucleico (in Spanish; at least among chemists). Nonetheless, it is worth noting that this is just one of the arguments, and the exact historical progression remains uncertain. Almost 100 years after the identification and naming of desoxyribose sugar and desoxyribonucleic acid, the term “desoxy” continues to see limited use even in the present times. We hope that this history will be remembered, and whenever young researchers come across the sporadic occurrence of “desoxy” in any published text, they will not consider it as a mistake.

## Data Availability

Data used in this analysis have been procured from open-source databases such as Google Ngram Viewer and NCBI-PubMed.
